# Highly Sensitive Liquid Core Temperature Sensor Based on Multimode Interference Effects

**DOI:** 10.3390/s151026929

**Published:** 2015-10-23

**Authors:** Miguel A. Fuentes-Fuentes, Daniel A. May-Arrioja, José R. Guzman-Sepulveda, Miguel Torres-Cisneros, José J. Sánchez-Mondragón

**Affiliations:** 1 Photonics and Optical Physics Laboratory, Optics Department, INAOE, Puebla, Puebla 72000, Mexico; E-Mails: migue_yimi@hotmail.com (M.A.F.-F.); delta_dirac@hotmail.com (J.J.S.-M.); 2 Centro de Investigaciones en Optica, Unidad Aguascalientes, Prol. Constitución 607, Fracc. Reserva Loma Bonita, Aguascalientes, Ags. 20200, Mexico; 3 CREOL, The College of Optics and Photonics, University of Central Florida, Orlando, FL 32816, USA; E-Mail: r.guzman@knights.ucf.edu; 4 NanoBioPhotonics Group, DICIS, University of Guanajuato, Salamanca, Guanajuato 368850, Mexico; E-Mail: mtorres@ugto.mx

**Keywords:** fiber optic sensor, temperature sensor, multimode interference

## Abstract

A novel fiber optic temperature sensor based on a liquid-core multimode interference device is demonstrated. The advantage of such structure is that the thermo-optic coefficient (TOC) of the liquid is at least one order of magnitude larger than that of silica and this, combined with the fact that the TOC of silica and the liquid have opposite signs, provides a liquid-core multimode fiber (MMF) highly sensitive to temperature. Since the refractive index of the liquid can be easily modified, this allows us to control the modal properties of the liquid-core MMF at will and the sensor sensitivity can be easily tuned by selecting the refractive index of the liquid in the core of the device. The maximum sensitivity measured in our experiments is 20 nm/°C in the low-temperature regime up to 60 °C. To the best of our knowledge, to date, this is the largest sensitivity reported for fiber-based MMI temperature sensors.

## 1. Introduction

Temperature sensors are important elements in a wide variety of industrial and research applications. Although different technologies can be used to develop temperature sensors, it is well known that optical fiber temperature sensors (OFTS) exhibit superior characteristics such as real-time response, immunity to external electromagnetic interference, compactness and stability, simple fabrication and repeatability, and the capability to operate in harsh environments. Moreover, if the fiber and the sensor architecture are carefully selected, it is possible to perform measurements with both high sensitivity and high resolution within the temperature range of interest.

Spectrally operated OFTS, in which the features of the spectral response of the sensor are related to the physical variable of interest have been developed using fiber Bragg gratings (FBG), long period gratings (LPFG), specialty fibers such as D-shaped fibers and photonic crystal fibers, multimode fibers (MMF), and more recently multi-core fibers. FBG and LPFG have been implemented for different sensing applications for the last two decades. Grating-based temperature sensors are compact (*i.e.*, short interaction length) but the sensitivity reported is only of a few tenths of pm/°C (<100 pm/°C) in the low temperature regime for the case of bare gratings [1,2]. Even the most recent approaches, it is the resolution over large temperature ranges what has been improved, but the sensitivity remains low on the order of 12 pm/°C [3]. Nevertheless, there are some techniques that can be applied to enhance the sensitivity of grating-based sensors. For example, by chirping the grating, the sensitivity has been enhanced up to 150–300 pm/°C in the case of LPFG [4]. The grating can be also inscribed in fibers with special doping, such as hydrogen-free boron-germanium (B-Ge) co-doped fibers, in which case the sensitivity is significantly improved up to 3.4 nm/°C [5]. The latest is actually the largest sensitivity reported for grating-based temperature sensors to the best of the author’s knowledge.

In-line fiber interferometry has also been widely investigated for temperature sensing applications. Interferometry-based techniques are known to exhibit high sensitivities and compact architectures in most applications. Interestingly, in the case of temperature sensing applications, the sensitivities reported for these in-line fiber structures are in general very low regardless of the interferometric configuration, *i.e.*, Fabry-Perot, Fizeau, and Mach-Zehnder, with values less than 50 pm/°C for temperatures up to 200 °C [6,7,8,9] and on the order of 100 pm/°C for higher temperatures [10]. This interferometric approach was extended to highly birefringent fibers and the sensitivity improved up to 0.25 nm/°C [11]. Nevertheless, it was only recently that devices based on these approaches achieved sensitivities on the order of 9.9 nm/°C when operating based on the interference of multiple modes [12,13]. The highest sensitivity achieved in a Fabry-Perot configuration is 260.7 nm/°C, and was achieved via thermal expansion of the iron V-groove that holds the device [14]. Other approaches include sensing structures based on photonic crystal fibers (PCF). In this case the sensitivity remains very low, less than 100 pm/°C, regardless of the sensitivity-enhancing technique implemented such as tapering [15,16]. Moreover, the interaction length of the sensing element (*i.e.*, PCF) needs to be on the order of tenths of centimeters in order to induce appreciable changes in the spectral response.

Another option that has been widely investigated is the use of multimode fibers (MMF), which allows multimode-interference-based measurements in compact architectures. The sensitivity reported for MMF-based sensors remains below 50 pm/°C for low temperature sensing [17,18,19,20], and on the order of 100 pm/°C for high temperature applications [21]. Similar sensitivities are obtained even if the interference is restricted to only a few modes by using graded-index MMF [18].

Recently, multi-core fibers (MCF) have attracted some attention for high-temperature sensing applications. However, similarly to other OFTS, their sensitivity remains very low. In this particular case the sensitivity reported is on the order of 50 pm/°C regardless of the number of cores [22,23].

An intrinsic issue, regardless of the technique and the architecture of the OFTS, is that they exhibit low sensitivities. This is correlated with the fact that the temperature changes are measured through the thermo-optic coefficient (TOC) of the silica which is in general very low, *i.e.*, on the order of 1 × 10^−5^ °C^−1^. Therefore, by allowing the fiber modes to interact with a material that exhibits a larger TOC should provide an OFTS with a higher sensitivity. For instance, a temperature sensor based on MMI effects achieved a sensitivity of −3.195 nm/°C by taking advantage of the TOC of the polymer coating of the MMF itself [24], and PCF-based temperature sensors infiltrated with either alcohol or index matching liquids have achieved sensitivities up to 42.818 nm/°C [25,26,27,28].

In this work, we propose and experimentally demonstrate a temperature sensor based on MMI effects that incorporates a liquid core MMF. The TOC of the liquid is at least one order of magnitude larger than that of silica and this, combined with the fact that the TOC of silica and the liquid have opposite signs, provides a liquid MMF highly sensitive to temperature. In addition, the availability of fluids with a wide range of refractive index allows us to control the modal properties of the liquid core MMF at will. Thus, we are able to tune both the sensor sensitivity and free-spectral range by simply selecting the appropriate refractive index of the liquid. We demonstrate MMI temperature sensors with sensitivities ranging from −0.46 nm/°C up to 20 nm/°C. To the knowledge of the authors, this is the largest sensitivity achieved in fiber optic MMI temperature sensors reported to date.

## 2. Multimode Interference Fiber Devices

**Figure 1:**
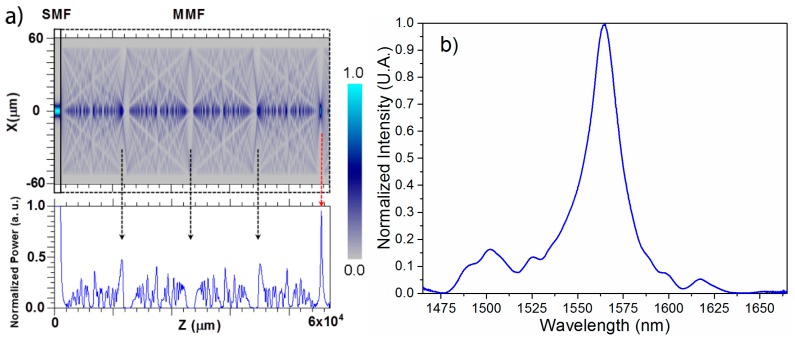
(**a**) Multimode interference phenomenon, (**b**) spectral response of MMI-based device.

The MMI phenomenon is inherent to multimode waveguides in which the excited modes interfere as they propagate along the MMF. As a result, an interference carpet is produced along the MMF and, at specific locations, self-images are produced and the input field is replicated. The formation of such self-images can be better observed using a beam propagation method (Beamprop from Rsoft®) as shown in Figure 1a. Rather than using the capillary fiber for Beamprop simulations, we use a standard 105/125 MMF to show that we do not require a special fiber in order to observe MMI effects. This is also convenient since the interference carpet is better observed for large core diameters. We would like to emphasize that this is a generic illustration of the MMI phenomena and the simulation parameters were chosen to provide a good visualization to the reader while keeping in mind that standard fibers are used. The 105/125 MMF has a core and cladding diameter of 105 µm and 125 µm, respectively. The operating wavelength is set to 1550 nm, and the refractive index of the core and cladding are 1.448 and 1.443, respectively. We can observe that at certain positions the light concentrates along the axis of the MMF, indicated by the arrows in Figure 1a, and they also exhibit a periodical behavior. This occurs at positions where the phase difference between the propagating modes is an integer multiple of 2π [29,30]. We can also notice that insertion losses are significantly reduced at the fourth self-image (red arrow) and this is the self-image that we typically use for MMI devices.

The self-image formation for MMI fiber devices has been widely investigated and is governed by the relation [29,30]:

(1) $$\lambda_{peak}=p\left(\frac{n_{MMF}W_{MMF}^{2}}{L}\right)$$

where *λ_peak_* is the wavelength at which the peak of the spectral response of the MMI device is expected in order to replicate the p-th image of the input field, *n_MMF_* and *W_MMF_* are the effective refractive index (RI) and the effective diameter of the MMF section, respectively, and *L* is the length of the MMF. The effective diameter *W_MMF_* is defined in terms of the nominal diameter of the MMF plus a factor that takes into account the portion of the light (evanescent tails) traveling in the cladding and is given by,

(2) WMMF=W+(λ0π)(nnc)2σ(nc2−n2)−12

where *λ_0_* is the free-space wavelength, *n_c_* and *n* are the refractive indexes of the core and cladding, respectively, and *W* is the nominal diameter of the MMF section (*i.e.*, physical diameter). The previous model was developed for planar waveguides and thus polarization effects are considered such that σ = 0 for TE polarization and σ = 1 for TM.

Using Equation (1), we can estimate the MMF physical length that will provide a specific peak wavelength, *i.e.*, the wavelength transmitted through the MMI device with negligible loss. However, when a broadband source is transmitted through a MMI device a bandpass spectral response is obtained, as shown in Figure 1b. This is due to the fact that, according to Equation (1), the different wavelengths will replicate the input field at different locations before/after the SMF-MMF interface and different amounts of power will be effectively coupled to the output SMF. The bandpass spectral response shown in Figure 1b corresponds to an all-fiber MMI device (with p = 4), and the response should be similar for integer multiples of p = 4. This type of wavelength response is convenient for sensing applications because we can follow either spectral shifts or intensity variations at a fixed wavelength, which can be correlated to the physical variable being measured.

## 3. Liquid Core MMI: Principle of Operation

In order to enhance the temperature sensitivity of a MMF we employ a capillary fiber filled with liquid. The advantage of such structure is that the thermo-optic coefficient (TOC) of the liquid is at least one order of magnitude larger than that of silica and this, combined with the fact that the TOC of silica and the liquid have opposite signs, provides a liquid-core multimode fiber (MMF) highly sensitive to temperature. Therefore, the peak wavelength response of the liquid-core MMI device as a function of temperature can be analytically obtained using Equation (1). As stated before, Equation (1) provides the expected peak wavelength that will replicate the p-th image of the input field in a MMI device of length *L* and optical parameters *n_MMF_* and *W_MMF_*. This equation also states that any change in the optical and/or the geometrical parameters of the MMF will shift the peak wavelength. Therefore, when thermal effects are considered, Equation (1) becomes

(3) $$\left(\lambda_{peak}+\Delta \lambda \right)=p\frac{\left(n_{MMF}+\Delta n\right){\left(W_{MMF}+\Delta W\right)}^{2}}{\left(L+\Delta L\right)}$$

where *ΔW* and *ΔL* relate to the thermal expansion of the MMF, *Δn* relates to the thermo-optic effect, and *Δλ* is the effective shift of the spectral response of the MMI device due to the thermal effects. For silica fibers, the thermal expansion effect (5 × 10^−7^ °C^−1^) is almost two orders of magnitude lower than the thermo-optic effect (~1 × 10^−5^ °C^−1^) and thus the overall effect can be attributed only to the thermo-optic contribution [31].

Even though thermal expansion could be neglected, the effective diameter still includes a thermo-optical component such that the overall spectral shift is determined either by both *n_MMF_* and *W_MMF_*, or their individual contribution, and is related to the refractive indexes of the core and cladding. When the MMF exhibits a large refractive index contrast between the core and the cladding the modes are strongly confined, and the effective diameter remains practically constant. This results in a linear dependence of the spectral shift on the effective refractive index that can be positive or negative depending on *Δn*. This means that the peak wavelength can shift to shorter or longer wavelengths depending on the sign of the thermo-optic coefficient. On the other hand, when the refractive index contrast is low the modes are loosely confined and then a quadratic behavior is expected for the spectral shift since the main contribution is from the effective diameter; this contribution is always positive and therefore we expect the spectral response to shift to longer wavelengths. Small refractive index contrast will involve contributions of both *n_MMF_* and *W_MMF_*.

The MMI spectral shift due to thermal effects was quantitatively evaluated using Equation (3) for a MMI device with a length of 25 mm and nominal diameter of 56 µm at p = 8. The coefficient of thermal expansion of the cladding was assumed to be α = 5 × 10^−7^ °C^−1^ and the core was assumed to have the same length of the cladding at all times. In order to replicate the liquid-core condition, the refractive index of the cladding was assumed to be that of silica (n = 1.443) and the refractive index of the core was evaluated from 1.46 to 1.60. The thermo-optic coefficient of silica was assumed to be ξ = 1 × 10^−5^ °C^−1^, which is in agreement with the values reported in the literature [1,5,20,22,24,32,33,34], and the thermo-optic coefficient of the liquid core was assumed to be ξ_c_ = −4 × 10^−4^ °C^−1^. The opposite sign together with the magnitude difference between the TOC of the core and the cladding leads to a finite temperature range in which the MMI can operate since the condition n_c_ > n must be satisfied in order for the guided modes to exist.

**Figure 2:**
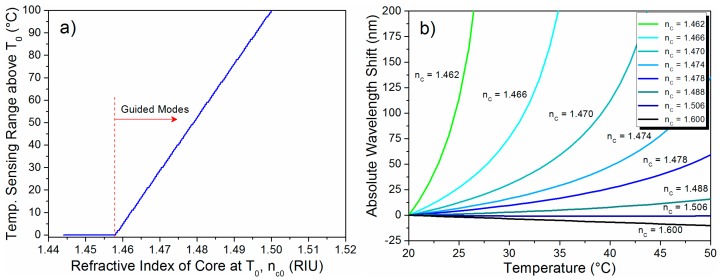
(**a**) Temperature sensing range in which guided modes exists; (**b**) Absolute wavelength shift of the MMI spectral response for different liquid core refractive index.

Figure 2a shows the temperature range available for sensing above the reference temperature T_0_ = 20 °C with the TOCs mentioned above. It can be seen that liquid cores with refractive index <1.46 at T_0_, even when the condition n_c_ > n is satisfied at T_0_, will not allow guided modes as the temperature is increased. Liquid cores with n_c_ around 1.46 at T_0_ will lead to temperature windows of only a few degrees before the modes stop being guided. Finally, liquid cores with refractive index >1.46 will allow mode guiding within a larger temperature range.

The absolute peak wavelength shift of the MMI device as a function of temperature for the parameters described above, as dictated by Equation (3), is shown in Figure 2b. The labels in the plot refer to the reference refractive index of the liquid core, at T_0_ = 20 °C, which was allowed to vary from 1.462 to 1.60. In our simulations, in order to get rid of polarization effects due to the circular symmetry of the fiber, the effective diameter was approximated using Equation (2) and averaging for both polarizations

(4) $$W_{MMF}=\frac{1}{2}\left(W_{MMF,TE}+W_{MMF,TM}\right)$$

As expected, the negative TOC of the liquid core leads to a negative linear response and a quadratic response for the case of strong and loose confinement, respectively. In other words, the spectral response of the MMI device shifts linearly to shorter wavelengths for high refractive index contrast while it shifts quadratically to longer wavelengths for the case of low refractive index contrast. Figure 2b confirms the reduction of the available sensing range as the refractive index of the core approaches that of the cladding. Nevertheless it also shows a dramatic increase in the spectral shift as a function of temperature, which greatly enhances the sensitivity of the sensor. Interestingly, using a liquid core with negative thermo-optic coefficient results in a transition from negative to positive spectral shift which in turn gives rise to a condition in which the spectral response of the MMI device remains practically invariant for certain temperature range. In this particular case this condition occurs at n_c_ ~ 1.5. This particular feature can be used to design temperature-insensitive *i.e.*, athermal MMI devices [31,35].

## 4. Liquid Core MMI: Experimental Results 

**Figure 3:**
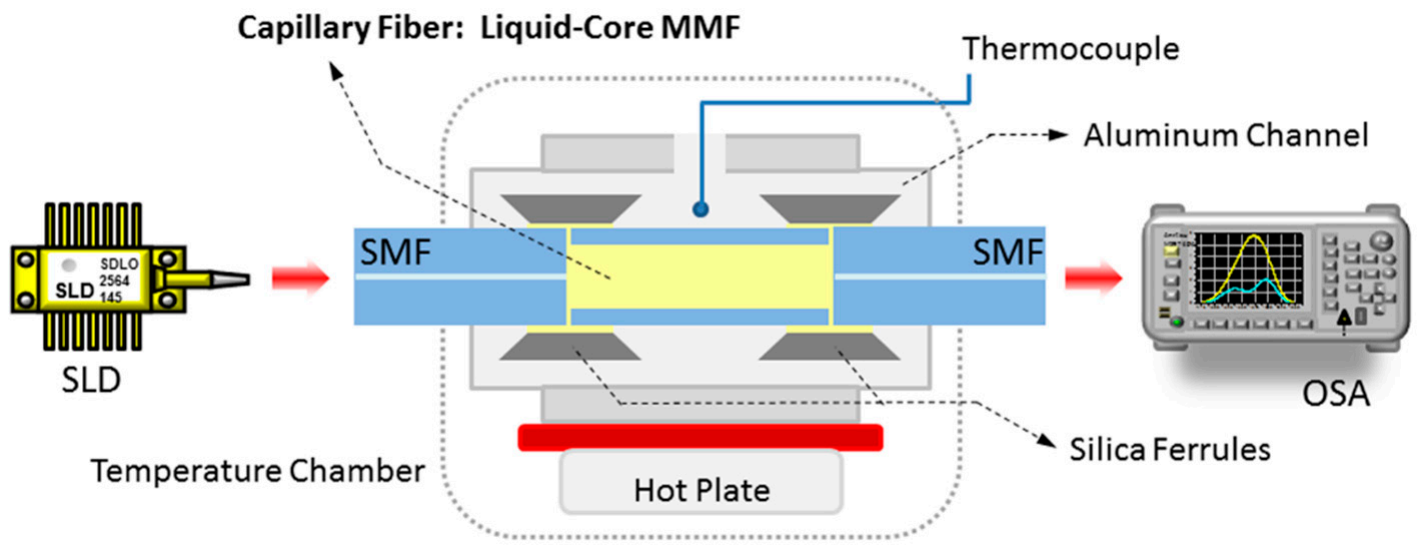
Schematic of the liquid-core temperature sensor based on MMI effects.

A schematic of the proposed fiber optic temperature sensor based on multimode interference effects is shown in Figure 3. The key element is the liquid-core MMF consisting of a capillary fiber with inner/outer diameter of 56/125 µm filled with index-matching oil (Cargille®, series A) with refractive index higher than that of the capillary (n = 1.443). According to the manufacturer, these oils have a thermo-optic coefficient of ξ_c_ ≈ −4 × 10^−4^ °C^−1^ for all the elements in the series, which is practically the same index considered in the simulations, and their stability is guaranteed up to 80 °C.

In order to evaluate the spectral response of the liquid-core MMI (LC-MMI) device, fused silica ferrules filled with the same RI liquid are used to match the input and output SMFs. It is worth mentioning that alignment is not a major concern since the inner diameter of the ferrule is 127 µm. Light from a broadband source, a super luminescent diode (SLD) with spectral width of ~200 nm centered at 1550 nm, is launched into the SMF–LC-MMF–SMF structure and the transmitted spectral response is measured with an optical spectrum analyzer (Anritsu MS9740A, Atsugi-shi, Japan) with a resolution of 0.5 nm.

**Figure 4:**
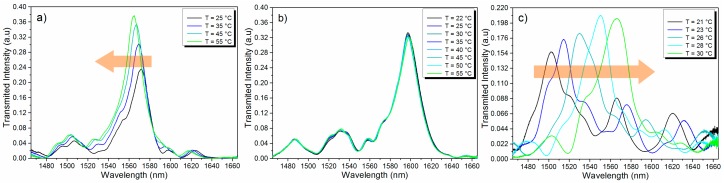
Experimental LC-MMI spectral response for (**a**) n_c0_ = 1.552, (**b**) n_c0_ = 1.510, and (**c**) n_c0_ = 1.464 at different temperatures.

Representative spectra of the experimental results are shown in Figure 4a–c. The set of spectra shown are for the three characteristic regions discussed above: the negative linear response, the athermal region, and the quadratic positive regime. As expected from the simulations, these regions are exhibited for reference refractive indices of the core of 1.552, 1.510, and 1.464, respectively. We should highlight that when we change the liquid refractive index we also change the capillary fiber length such that the initial and subsequent spectra fall into the spectral window of interest. This is easily achieved by cleaving different capillary fibers for each liquid. The change in the capillary fiber length can be considered as part of the sensor design as it allows defining the wavelength range in which the sensor will operate.

The insertion loss of the sensor ranges from 4.4 dB for liquids with high RI to 8.1 dB as the RI of liquid is reduced and is close to the RI value of the capillary fiber. Since a spliced MMI has a typical insertion loss of 0.5 dB and the liquid has an insertion loss of approximately 2.2 dB (0.088 dB/mm), we estimate that the loss due to misalignment is about 1.7 dB. When the RI of the liquid is reduced higher losses are induced due to the reduced confinement of the propagating modes, and reaches its maximum of 8.1 dB when the liquid RI is close to that of the capillary fiber. Insertion losses could be reduced using an adequate polymer with lower absorption losses, and also a ferrule with inner diameter close to 125 µm.

**Figure 5:**
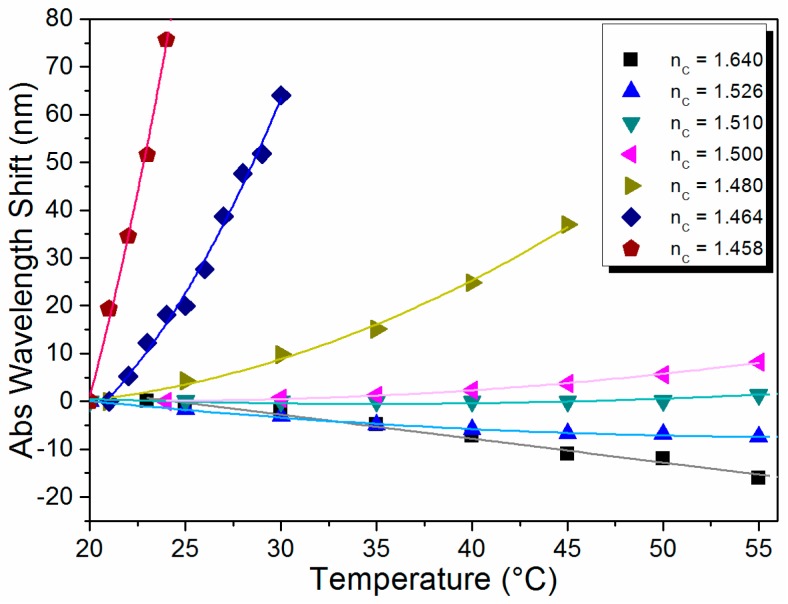
Absolute wavelength shift measured experimentally as a function of temperature for different liquid core refractive indexes.

The absolute peak wavelength shift as a function of temperature for the different oils experimentally evaluated is shown in Figure 5. We can easily observe that the liquid cores with higher RI confine the modes more strongly and, therefore, the spectral response shifts linearly with a negative slope, *i.e.*, to shorter wavelengths. On the other hand, the liquids with lower RI values shift to longer wavelengths and exhibit a quadratic dependence on temperature. The experimental results confirm the tradeoff between the regime of the sensor response (linear or quadratic), the sensor sensitivity, and the temperature range (free-spectral range) over which the sensor operates: the higher the sensitivity the smaller the sensing range. Choosing the right RI allows selecting both the sensor sensitivity and type of response.

The temperature range explored in the experiments was restricted in order to preserve the integrity of the index-matching oils. However, the same modeling and approach can be used for any other material that could potentially have more adequate thermal and optical properties. For instance, polymeric materials exhibit TOC on the same order as the index-matching liquids but they have the advantage that the temperature range can be significantly extended without compromising the integrity of the core material. Moreover, given the characteristics and particular features of the SMF–LC-MMF–SMF architecture, it could be used as the basis of temperature-insensitive devices and could be easily included in applications related to thermally-tuned lasers.

## 5. Conclusions

In summary, a novel fiber optic temperature sensor based on a liquid core MMI device was demonstrated. The fact that we have a wide range of refractive index liquids combined with the mode properties of the liquid-core MMF allows us to control both the sensitivity and the free spectral range of the sensor by simply selecting the refractive index of the liquid section. A maximum sensitivity of 20 nm/°C is achieved in our experiments and, as far as we know, this is the largest sensitivity reported to date for fiber-based MMI temperature sensors. We also identified a particular refractive index value that makes the MMI device temperature insensitive, which can be used for wavelength locking and applications where temperature stability is critical.
